# ISO 15189:2012: What changes for African laboratories?

**DOI:** 10.4102/ajlm.v4i1.325

**Published:** 2015-07-31

**Authors:** Nicolas Bouchet

**Affiliations:** 1 Quality Assurance Department, Centre National de Recherche et de Formation sur le Paludisme (CNRFP), Burkina Faso

## Introduction

The International Organization for Standardization's (ISO) 15189 is the gold standard quality criteria for medical laboratories^1^ and accreditation is the recognition of a quality system in full compliance with this standard. Aware of the current challenges in the fields of public health and biomedical research, African medical laboratories may need to develop Quality Management Systems (QMS) compliant with the requirements of ISO 15189. Launched by the World Health Organization's Regional Office for Africa (WHO AFRO) and partners, the Stepwise Laboratory Quality Improvement Process Towards Accreditation (SLIPTA) and Strengthening Laboratory Management Toward Accreditation (SLMTA) programmes have emerged in recent years in Africa to support laboratories in the implementation of quality processes.^2,3,4,5^

As with any other quality system, ISO standards evolve and are revised periodically. At the end of 2012, ISO published the third edition of ISO 15189,^6^ which replaces the 2007 version.^7^ The goal of this article is to highlight the major changes between the two versions of this standard, as well as the modifications these changes will require in quality systems already in place in laboratories, especially in African laboratories.

## ISO 15189:2012

In the new version, the standard is a clear document which is better organised than the 2007 version. The title was shortened and the organisation of the different sections has been adjusted to follow a more logical order, allowing users to gain a better understanding of the different requirements. During the presentation of the new version, ISO spoke of a ‘technical revision’; however, a reading of this new version reveals changes that are actually more substantial.

### Process approach

A process is ‘a set of interrelated or interacting activities that transform inputs into output elements’.^8^ The process approach describes the organisation of work as a series of interconnected steps with the goal of meeting optimal customer satisfaction and achievement of objectives. The goal is to make the organisation more functional and less dependent on hierarchy, with the introduction and development of cross-functional management, a result-oriented culture and teamwork within the laboratory.

The 2007 version already addressed this concept, but left the choice open to laboratories as to whether or not to follow this approach in their QMS. The process approach already existed in the QMS based on the general quality standard, ISO 9001, which introduced the concept in its 2000 version^9^ and maintained it in the 2008 version.^10^ The 2012 version of ISO 15189 is much more explicit: the process approach has become a requirement (subsection 4.2.1), albeit one that could be complicated to implement for African laboratories.

In fact, this approach may be difficult to implement for any small medical laboratory with a small staff; such laboratories represent the majority of medical laboratories in sub-Saharan Africa. The process approach involves the mapping of the effect of processes, that is, to represent all the processes needed for the QMS and their sequences by determining the interactions that bind them, and appointing process managers with clearly-delineated role definitions. In small organisations, the same person may have both an overall and a detailed view of operations, thus a precise mapping of different processes is often seen as unnecessary. For example, if in a laboratory of four employees each individual is involved in all processes, the establishment of a map may seem superfluous.

However, this approach does have its advantages. Firstly, it emphasises the ultimate goal of the QMS – patient outcomes – by defining and clearly showing each staff member's role in the achievement of these outcomes. Secondly, it allows analysis (and thus improvement) of the performance of the laboratory.

Medical laboratories have an advantage an implementing this approach, since their activities are centred around three main processes defined in the standard: the preanalytical process (Section 5.4), the analytical process (Section 5.5) and the postanalytical process (Section 5.7). By focusing on these three major processes, a laboratory may be able to link to other processes (such as equipment and inventory management, human resources, customer satisfaction, continuous improvement, etc.) for a fairly simple mapping.

### Quality manual

For the quality manual, the 2007 version (subsection 4.2.4) proposed a plan in 23 points; the 2012 version focuses on describing six key points in a laboratory quality manual (subparagraph 4.2.2.2): the quality policy, a description of the scope of the QMS, the organisation and the structure of the laboratory, the roles and responsibilities of laboratory management, the description of the document management system and the policies established for the QMS, with reference to the activities that support them.

This change will thus allow each laboratory to define the terms of its quality manual based on its own quality system, which will demonstrate mastery of the standard by the laboratory and the ability of the laboratory to adapt the standard to local realities, which often proves difficult in sub-Saharan Africa. It will also simplify the writing of this document, which is the cornerstone of the QMS.

### Risk management

This is arguably the major change in the new version of the standard. It appears in subparagraph 4.1.1.4, where it is stated that the role of the laboratory director is to ‘design and implement a contingency plan’ and that these plans should be tested periodically. Subsection 4.14.6 (Risk management) also addresses this subject, focusing on the risks of possible failures on the test results; everything must be done to reduce and/or eliminate these risks. The concept is also found in the new section on the management of laboratory information (subsection 5.10.3); it is stated in this paragraph that ‘the laboratory shall have documented contingency plans […] in the event of failure or downtime’.

This concept of risk management is an important introduction in this new version of the standard; it will require substantial work for its implementation, as all contingencies should be identified and evaluated (e.g. stock-outs of reagents caused by delivery delays, failure of an analyser, power failure, malfunction of a printer, etc.). Each situation must be included in the ‘contingency plan’ with:

- A solution to the problem (correction).
- A method to inform both patients and physicians.
- A plan to communicate the problem internally.
- Corrective actions to prevent the recurrence of the problem, and
- Procedures/protocols to return to normal operations.

These plans must then be ‘tested periodically’ and this process must be documented; these tests should then be used to improve the plan if necessary. These plans will also be audited as part of an internal audit of the QMS.

Let us take as examples power failures and delays in supply of reagents. These are rare events in industrialised countries, but are common problems in sub-Saharan Africa. Many African capitals still experience rolling blackouts during certain periods of the year and delivery delays are common for laboratory reagents. A risk with a very low probability of occurrence in industrialised countries may occur with a very high frequency in sub-Saharan African countries. It could thus be difficult to implement risk management as described in the standard in African laboratories. Contingency plans can be implemented periodically for some aspects, but some things that might otherwise be considered ‘exceptional’ may need to be considered routine [in a sub-Saharan setting].

It should be noted that this introduction of the concept of risk management in the new version of ISO 15189 is part of a more global trend. This concept, which comes from industry, has entered the regulatory world in recent years. In the process of revision of the ISO 9001 standard, which is the basic reference for quality management systems (the new version is scheduled for 2015), the concept of a risk management approach was identified by the expert group in charge of the revision of ISO 9001 during the 26th meeting of the ISO Technical Committee in Tokyo in 2009^11^ and it will be integrated into the new version. Risk management is clearly one of the objectives of this new version of ISO 9001, requiring one to project into the future to anticipate all possible problems that could prevent customer satisfaction, which is the main objective of the standard. In the field of medical laboratories, this concept was introduced in the United States by the new regulations in Quality Control, with the Internal Quality Control Plan, following the Clinical and Laboratory Standards Institute (CLSI) EP23-A guideline.^12^ This document introduced the concept of risk management in the field of internal quality control in medical laboratories.^13,14,15^

### Laboratory information management

Section 5.10 is not really new in the new version of the standard; it is rather Annex B of the 2007 version, which has now become a normative, with the obligation to implement these requirements. This entire section focuses on data protection and the management of the information system. The laboratory must ensure that the software tools involved in managing laboratory information (collection, processing, recording, reporting, data retention) are validated by suppliers, regularly maintained and secure. Laboratories must consider these new requirements and act accordingly, although they may be difficult to implement and to document in the African context.

### Minor changes

Section 4.14, which was dedicated only to internal audits in the 2007 version, is more complete in the 2012 version, because it deals with all aspects of audits and evaluations: review of requests, procedures and requirements for samples, assessment of customer feedback, staff suggestions, internal and external audits, risk management and monitoring of quality indicators. The goal of this entire process of evaluation/audit is to improve the laboratory quality system, ensuring that all quality processes necessary to ensure the best quality for patients’ results have been implemented in order to meet customer requirements.

A new subparagraph (4.1.1.3) introduces ethics rules about potential conflicts of interest, the integrity of staff, ethical considerations in handling samples of human origin and confidentiality of patients. This point is very important because the culture of business ethics is a concept seldom implemented in sub-Saharan Africa, contrary to the concept of bioethics, which was mentioned in the 2007 version (Annex C), but has disappeared in the 2012 version.

## Conclusion

ISO 15189 remains the ‘gold standard’ for quality in medical laboratories. The cleaner organisation of the 2012 version will allow laboratories to better understand this standard and better meet the standard requirements. In fact, it is an obligation for laboratories to use the ISO standard to move toward accreditation in order to ensure the trust of patients and to gain national and international respect. The challenge is huge for African laboratories, which must adapt to this standard whilst taking into account the specific conditions in which they are based. It is desirable that WHO AFRO adapts its SLIPTA checklist to this new version of the standard, generally adapting all support programmes to include the changes of the 2012 version.

## References

[1] Datema TAM, Oskam L, Klatser PR. Review and comparison of quality standards, guidelines and regulations for laboratories. Afr J Lab Med. 2011;1(1), Art.#3, 7 pages. 10.4102/ajlm.v1i1.3 PMC5644525 29062723

[2] Gershy-Damet G, Rotz P, Cross D. Belabbes E, Cham F, Ndihokubwayo J, et al. The World Health Organization African Region Laboratory Accreditation Process. Am J Clin Pathol. 2010; 134:393-400. 10.1309/AJCPTUUC2V1WJQBM 20716795

[3] Maruta T, Motebang D, Wanyoike J, Peter T, Rotz PJ. Impact of mentorship on WHO-AFRO Strengthening Laboratory Quality Improvement Process Towards Accreditation (SLIPTA). Afr J Lab Med. 2012;1(1), Art. #6, 8 pages. 29062726 10.4102/ajlm.v1i1.6 PMC5644515

[4] Yao K, McKinney B, Murphy A, Rotz P, Wafula W, Sendagire H, Okui S, et al. Improving Quality Management Systems of Laboratories in Developing Countries. Am J Clin Pathol. 2010;134:401-409. 10.1309/AJCPNBBL53FWUIQJ 20716796

[5] Mothabeng D, Maruta T, Lebina M, Lewis K, Wanyoike J, Mengstu Y. Strengthening Laboratory Management Towards Accreditation: The Lesotho experience. Afr J Lab Med. 2012;1(1), Art. #9, 7 pages. 29062729 10.4102/ajlm.v1i1.9 PMC5644518

[6] Organisation Internationale de Normalisation (ISO). ISO 15189 : 2012. Laboratoires de biologie médicale – Exigences concernant la qualité et la compétence. Troisième édition 2012-11-01, Version corrigée 2013-03-01.

[7] Organisation Internationale de Normalisation (ISO). ISO 15189 : 2007. Laboratoires d’analyses de biologie médicale – Exigences particulières concernant la qualité et la compétence. Deuxième édition 2007-04-15, Version corrigée 2007-09-15.

[8] Site internet de l’Organisation Internationale de Normalisation (ISO). Disponible à cette adresse: www.iso.org

[9] Organisation Internationale de Normalisation (ISO). ISO 9000 : 2005 Systèmes de management de la qualité – Principes essentiels et vocabulaire. Troisième édition 2005-09-15.

[10] Organisation Internationale de Normalisation (ISO). ISO 9001 : 2000 Systèmes de management de la qualité – Exigences. Troisième édition 2000-12-15.

[11] Organisation Internationale de Normalisation (ISO). ISO 9001 : 2008 Systèmes de management de la qualité – Exigences. Quatrième édition 2008-11-15, Version corrigée 2009-07-15.

[12] Clinical and Laboratory Standards Institute. EP23-A: *Laboratory Quality Control Based on Risk Management; Approved Guideline*. 2011.

[13] Person N. Developing Risk-based Quality Control Plans: An Overview of CLSI EP23-A. Clin Lab Med, 2013, 33, 15-26. 10.1016/j.cll.2012.11.003 23331726

[14] Westgard J. Perspectives on Quality Control, Risk Management, and Analytical Quality Management. Clin Lab Med, 2013, 33, 1-14. 10.1016/j.cll.2012.10.003 23331725

[15] Nichols J. Laboratory Quality Control Based on Risk Management. Ann Saudi Med. 2011 May-Jun; 31(3): 223–228. 10.4103/0256-4947.81526 21623049 PMC3119960

